# Supplementary material for: The reciprocal relationships between social media self-control failure, mindfulness and wellbeing: A longitudinal study
Source: PLoS One. 2021 Aug 4;16(8):e0255648. doi: 10.1371/journal.pone.0255648 (PMC8336798; doi:10.1371/journal.pone.0255648)
Supplement: S2 File — (DOCX) [file pone.0255648.s002.docx]

**S1 File. Results including control variables**

| **Table 1. Reciprocal relationships between social media self-control failure and mindfulness adding age as a control variable** | | | | | | | | | | | | | |
| --- | --- | --- | --- | --- | --- | --- | --- | --- | --- | --- | --- | --- | --- |
|  | | Unconstrained model (*df = 9*) | | | Constrained autoregressive paths (*df* = 7) | | | Constrained cross-lagged paths (*df* = 7) | | | Constrained autoregressive and cross-lagged paths (*df* = 9) | | |
|  | | *b* | *SE* | *p* | *b* | *SE* | *p* | *b* | *SE* | *p* | *b* | *SE* | *p* |
| Autoregressive paths | | | | | | | | | | | | | |
| SMSCF T1 → SMSCF T2 | | -0.083 | 0.113 | 0.463 | -0.038 | 0.095 | 0.691 | -0.120 | 0.111 | 0.283 | -0.037 | 0.105 | 0.727 |
| SMSCF T2 → SMSCF T3 | | 0.032 | 0.118 | 0.789 | -0.038 | 0.095 | 0.691 | 0.075 | 0.111 | 0.496 | -0.037 | 0.105 | 0.727 |
| MF T1 → MF T2 | | 0.262 | 0.198 | 0.185 | 0.404 | 0.160 | **0.012^*^** | 0.319 | 0.198 | 0.107 | 0.386 | 0.149 | **0.009^**^** |
| MF T2 → MF T3 | | 0.433 | 0.127 | **0.001^**^** | 0.404 | 0.160 | **0.012^*^** | 0.398 | 0.136 | **0.004^**^** | 0.386 | 0.149 | **0.009^**^** |
| Cross-lagged paths | | | | | | | | | | | | | |
| SMSCF T1 → MF T2 | -0.163 | | 0.079 | **0.038^*^** | -0.173 | 0.063 | **0.006^**^** | -0.112 | 0.058 | 0.055 | -0.136 | 0.063 | **0.031^*^** |
| SMSCF T2 → MF T3 | -0.031 | | 0.086 | 0.722 | -0.075 | 0.099 | 0.446 | -0.112 | 0.058 | 0.055 | -0.136 | 0.063 | **0.031^*^** |
| MF T1 → SMSCF T2 | -0.104 | | 0.227 | 0.647 | -0.228 | 0.187 | 0.222 | -0.295 | 0.162 | 0.068 | -0.372 | 0.178 | **0.037^*^** |
| MF T2 → SMSCF T3 | -0.456 | | 0.180 | **0.011^*^** | -0.499 | 0.183 | **0.006^**^** | -0.295 | 0.162 | 0.068 | -0.372 | 0.178 | **0.037^*^** |
| Covariance | | | | | | | | | | | | | |
| Between-person | -0.249 | | 0.047 | **0.000^***^** | -0.228 | 0.047 | **0.000^***^** | -0.244 | 0.136 | 0.072 | -0.228 | 0.048 | **0.000^***^** |
| Intra-wave correlation T1 | -0.062 | | 0.043 | 0.148 | -0.079 | 0.040 | **0.047^*^** | -0.064 | 0.134 | 0.633 | -0.079 | 0.039 | **0.044^*^** |
| Intra-wave correlation T2 | -0.096 | | 0.038 | **0.011^*^** | -0.115 | 0.039 | **0.003^**^** | **-**0.100 | 0.039 | **0.010^*^** | -0.115 | 0.044 | **0.009^**^** |
| Intra-wave correlation T3 | -0.051 | | 0.019 | **0.009^**^** | -0.054 | 0.020 | **0.007^**^** | -0.048 | 0.019 | **0.011^*^** | -0.048 | 0.019 | **0.012^*^** |
| Control variables |  | |  |  |  |  |  |  |  |  |  |  |  |
| SMSCF T1→age | -0.026 | | 0.004 | **0.000^***^** | -0.026 | 0.004 | **0.000^***^** | -0.026 | 0.004 | **0.000^***^** | -0.026 | 0.004 | **0.000^***^** |
| SMSCF T2→age | -0.026 | | 0.004 | **0.000^***^** | -0.026 | 0.004 | **0.000^***^** | -0.026 | 0.004 | **0.000^***^** | -0.026 | 0.004 | **0.000^***^** |
| SMSCF T3 → age | -0.026 | | 0.004 | **0.000^***^** | -0.026 | 0.004 | **0.000^***^** | -0.026 | 0.004 | **0.000^***^** | -0.026 | 0.004 | **0.000^***^** |
| MF T1 → age | 0.013 | | 0.003 | **0.000^***^** | 0.014 | 0.003 | **0.000^***^** | 0.014 | 0.003 | **0.000^***^** | 0.014 | 0.003 | **0.000^***^** |
| MF T2 → age | 0.013 | | 0.003 | **0.000^***^** | 0.014 | 0.003 | **0.000^***^** | 0.014 | 0.003 | **0.000^***^** | 0.014 | 0.003 | **0.000^***^** |
| MF T3 → age | 0.013 | | 0.003 | **0.000^***^** | 0.014 | 0.003 | **0.000^***^** | 0.014 | 0.003 | **0.000^***^** | 0.014 | 0.003 | **0.000^***^** |
| *N*_T1_ = 594, *N*_T2_ = 410, *N*_T3_ = 329, SMSCF = Social media self-control failure, MF = Mindfulness.  ^***^*p* < .001, ^**^*p* < .01, ^*^*p* < .05 | | | | | | | | | | | | | |

| **Table 2. Reciprocal relationships between social media self-control failure and mindfulness adding sex as a control variable** | | | | | | | | | | | | | |
| --- | --- | --- | --- | --- | --- | --- | --- | --- | --- | --- | --- | --- | --- |
|  | | Unconstrained model (*df = 9*) | | | Constrained autoregressive paths (*df* = 7) | | | Constrained cross-lagged paths (*df* = 7) | | | Constrained autoregressive and cross-lagged paths (*df* = 9) | | |
|  | | *b* | *SE* | *p* | *b* | *SE* | *p* | *b* | *SE* | *p* | *b* | *SE* | *p* |
| Autoregressive paths | | | | | | | | | | | | | |
| SMSCF T1 → SMSCF T2 | | -0.092 | 0.103 | 0.373 | -0.073 | 0.092 | 0.429 | -0.122 | 0.106 | 0.249 | -0.075 | 0.102 | 0.464 |
| SMSCF T2 → SMSCF T3 | | -0.029 | 0.130 | 0.823 | -0.073 | 0.092 | 0.429 | 0.023 | 0.129 | 0.860 | -0.075 | 0.102 | 0.464 |
| MF T1 → MF T2 | | 0.232 | 0.205 | 0.257 | 0.394 | 0.176 | **0.025^*^** | 0.282 | 0.206 | 0.171 | 0.380 | 0.170 | **0.026^*^** |
| MF T2 → MF T3 | | 0.423 | 0.130 | **0.001^**^** | 0.394 | 0.176 | **0.025^*^** | 0.389 | 0.145 | **0.007^**^** | 0.380 | 0.170 | **0.026^*^** |
| Cross-lagged paths | | | | | | | | | | | | | |
| SMSCF T1 → MF T2 | -0.140 | | 0.076 | 0.065 | -0.140 | 0.063 | **0.027** | -0.091 | 0.061 | 0.135 | -0.106 | 0.067 | 0.110 |
| SMSCF T2 → MF T3 | -0.012 | | 0.092 | 0.893 | -0.055 | 0.110 | 0.618 | -0.091 | 0.061 | 0.135 | -0.106 | 0.067 | 0.110 |
| MF T1 → SMSCF T2 | -0.061 | | 0.218 | 0.778 | -0.178 | 0.187 | 0.339 | -0.246 | 0.167 | 0.140 | -0.300 | 0.179 | 0.094 |
| MF T2 → SMSCF T3 | -0.425 | | 0.182 | **0.019^*^** | -0.433 | 0.186 | **0.020^*^** | -0.246 | 0.167 | 0.140 | -0.300 | 0.179 | 0.094 |
| Covariance | | | | | | | | | | | | | |
| Between-person | -0.284 | | 0.044 | **0.000^***^** | -0.267 | 0.047 | **0.000^***^** | -0.280 | 13.738 | 0.984 | -0.267 | 0.048 | **0.000^***^** |
| Intra-wave correlation T1 | -0.064 | | 0.039 | 0.100 | -0.079 | 0.038 | **0.038^*^** | -0.067 | 13.738 | 0.996 | -0.081 | 0.037 | **0.031^*^** |
| Intra-wave correlation T2 | -0.080 | | 0.037 | **0.032^*^** | -0.097 | 0.041 | **0.018^*^** | -0.083 | 0.041 | **0.046^*^** | -0.094 | 0.046 | **0.041^*^** |
| Intra-wave correlation T3 | -0.048 | | 0.020 | **0.018^*^** | -0.050 | 0.021 | **0.018^*^** | -0.044 | 0.019 | **0.021^*^** | -0.044 | 0.020 | **0.026^*^** |
| Control variables |  | |  |  |  |  |  |  |  |  |  |  |  |
| SMSCF T1 → sex | 0.240 | | 0.085 | **0.005^**^** | 0.238 | 0.085 | **0.005^**^** | 0.239 | 0.085 | **0.005^**^** | 0.238 | 0.085 | **0.005^**^** |
| SMSCF T2 → sex | 0.240 | | 0.085 | **0.005^**^** | 0.238 | 0.085 | **0.005^**^** | 0.239 | 0.085 | **0.005^**^** | 0.238 | 0.085 | **0.005^**^** |
| SMSCF T3 → sex | 0.240 | | 0.085 | **0.005^**^** | 0.238 | 0.085 | **0.005^**^** | 0.239 | 0.085 | **0.005^**^** | 0.238 | 0.085 | **0.005^**^** |
| MF T1 → sex | -0.091 | | 0.071 | 0.200 | -0.086 | 0.071 | 0.229 | -0.085 | 0.071 | 0.233 | -0.081 | 0.071 | 0.254 |
| MF T2 → sex | -0.091 | | 0.071 | 0.200 | -0.086 | 0.071 | 0.229 | -0.085 | 0.071 | 0.233 | -0.081 | 0.071 | 0.254 |
| MF T3 → sex | -0.091 | | 0.071 | 0.200 | -0.086 | 0.071 | 0.229 | -0.085 | 0.071 | 0.233 | -0.081 | 0.071 | 0.254 |
| *N*_T1_ = 594, *N*_T2_ = 410, *N*_T3_ = 329, SMSCF = Social media self-control failure, MF = Mindfulness.  ^***^*p* < .001, ^**^*p* < .01, ^*^*p* < .05 | | | | | | | | | | | | | |

| **Table 3. Reciprocal relationships between social media self-control failure and mindfulness adding frequency of visit on social media as a control variable** | | | | | | | | | | | | | |
| --- | --- | --- | --- | --- | --- | --- | --- | --- | --- | --- | --- | --- | --- |
|  | | Unconstrained model (*df = 9*) | | | Constrained autoregressive paths (*df* = 7) | | | Constrained cross-lagged paths (*df* = 7) | | | Constrained autoregressive and cross-lagged paths (*df* = 9) | | |
|  | | *b* | *SE* | *p* | *b* | *SE* | *p* | *b* | *SE* | *p* | *b* | *SE* | *p* |
| Autoregressive paths | | | | | | | | | | | | | |
| SMSCF T1 → SMSCF T2 | | -0.085 | 0.106 | 0.421 | -0.046 | 0.098 | 0.637 | -0.113 | 0.105 | 0.278 | -0.049 | 0.105 | 0.638 |
| SMSCF T2 → SMSCF T3 | | 0.028 | 0.124 | 0.819 | -0.046 | 0.098 | 0.637 | 0.060 | 0.120 | 0.615 | -0.049 | 0.105 | 0.638 |
| MF T1 → MF T2 | | 0.229 | 0.202 | 0.256 | 0.383 | 0.177 | **0.030^*^** | 0.273 | 0.203 | 0.178 | 0.370 | 0.165 | **0.025^*^** |
| MF T2 → MF T3 | | 0.414 | 0.133 | **0.002^**^** | 0.383 | 0.177 | **0.030^*^** | 0.384 | 0.142 | **0.007^**^** | 0.370 | 0.165 | **0.025^*^** |
| Cross-lagged paths | | | | | | | | | | | | | |
| SMSCF T1 → MF T2 | -0.139 | | 0.074 | 0.061 | -0.145 | 0.062 | **0.019^*^** | -0.094 | 0.058 | 0.102 | -0.115 | 0.064 | 0.071 |
| SMSCF T2 → MF T3 | -0.022 | | 0.089 | 0.800 | -0.063 | 0.107 | 0.560 | -0.094 | 0.058 | 0.102 | -0.115 | 0.064 | 0.071 |
| MF T1 → SMSCF T2 | -0.102 | | 0.218 | 0.641 | -0.214 | 0.109 | 0.261 | -0.254 | 0.164 | 0.121 | -0.321 | 0.180 | 0.074 |
| MF T2 → SMSCF T3 | -0.386 | | 0.188 | **0.041^*^** | -0.423 | 0.204 | **0.038^*^** | -0.254 | 0.164 | 0.121 | -0.321 | 0.180 | 0.074 |
| Covariance | | | | | | | | | | | | | |
| Between-person | -0.251 | | 0.043 | **0.000^***^** | -0.232 | 0.062 | **0.000^***^** | -0.248 | 0.348 | 0.476 | -0.232 | 0.047 | **0.000^***^** |
| Intra-wave correlation T1 | -0.066 | | 0.039 | 0.092 | -0.083 | 0.056 | 0.139 | 0.067 | 0.347 | 0.848 | -0.082 | 0.038 | **0.032^*^** |
| Intra-wave correlation T2 | -0.081 | | 0.038 | **0.031^*^** | -0.099 | 0.043 | **0.020^*^** | -0.086 | 0.039 | **0.030^*^** | -0.100 | 0.045 | **0.027^*^** |
| Intra-wave correlation T3 | -0.046 | | 0.020 | **0.021^*^** | -0.048 | 0.021 | **0.024^*^** | **-0.044^*^** | 0.019 | **0.019^*^** | -0.044 | 0.020 | **0.025^*^** |
| Control variables |  | |  |  |  |  |  |  |  |  |  |  |  |
| SMSCF T1 → frequency | 0.289 | | 0.031 | **0.000^***^** | 0.288 | 0.031 | **0.000^***^** | 0.291 | 0.031 | **0.000^***^** | 0.289 | 0.031 | **0.000^***^** |
| SMSCF T2 → frequency | 0.289 | | 0.031 | **0.000^***^** | 0.288 | 0.031 | **0.000^***^** | 0.291 | 0.031 | **0.000^***^** | 0.289 | 0.031 | **0.000^***^** |
| SMSCF T3 → frequency | 0.289 | | 0.031 | **0.000^***^** | 0.288 | 0.031 | **0.000^***^** | 0.291 | 0.031 | **0.000^***^** | 0.289 | 0.031 | **0.000^***^** |
| MF T1 → frequency | -0.088 | | 0.030 | **0.003^**^** | -0.088 | 0.030 | **0.003^**^** | -0.089 | 0.030 | **0.003^**^** | -0.089 | 0.030 | **0.003^**^** |
| MF T2 → frequency | -0.088 | | 0.030 | **0.003^**^** | -0.088 | 0.030 | **0.003^**^** | -0.089 | 0.030 | **0.003^**^** | -0.089 | 0.030 | **0.003^**^** |
| MF T3→frequency | -0.088 | | 0.030 | **0.003^**^** | -0.088 | 0.030 | **0.003^**^** | -0.089 | 0.030 | **0.003^**^** | -0.089 | 0.030 | **0.003^**^** |
| *N*_T1_ = 594, *N*_T2_ = 410, *N*_T3_ = 329, SMSCF = Social media self-control failure, MF = Mindfulness.  ^***^*p* < .001, ^**^*p* < .01, ^*^*p* < .05 | | | | | | | | | | | | | |

| **Table 4. Reciprocal relationships between social media self-control failure and mindfulness adding time spent on social media as a control variable** | | | | | | | | | | | | | |
| --- | --- | --- | --- | --- | --- | --- | --- | --- | --- | --- | --- | --- | --- |
|  | | Unconstrained model (*df = 9*) | | | Constrained autoregressive paths (*df = 7*) | | | Constrained cross-lagged paths (*df = 7*) | | | Constrained autoregressive and cross-lagged paths (*df = 9*) | | |
|  | | *b* | *SE* | *p* | *b* | *SE* | *p* | *b* | *SE* | *p* | *b* | *SE* | *p* |
| Autoregressive paths | | | | | | | | | | | | | |
| SMSCF T1 → SMSCF T2 | | -0.133 | 0.107 | 0.211 | -0.080 | 0.098 | 0.415 | -0.157 | 0.107 | 0.143 | -0.086 | 0.107 | 0.421 |
| SMSCF T2 → SMSCF T3 | | 0.030 | 0.126 | 0.811 | -0.080 | 0.098 | 0.415 | 0.060 | 0.122 | 0.620 | -0.086 | 0.107 | 0.421 |
| MF T1 → MF T2 | | 0.231 | 0.204 | 0.259 | 0.389 | 0.182 | **0.033^*^** | 0.262 | 0.208 | 0.207 | 0.373 | 0.175 | **0.033^*^** |
| MF T2 → MF T3 | | 0.412 | 0.135 | **0.002^**^** | 0.389 | 0.182 | **0.033^*^** | 0.388 | 0.147 | **0.008^**^** | 0.373 | 0.175 | **0.033^*^** |
| Cross-lagged paths | | | | | | | | | | | | | |
| SMSCF T1 → MF T2 | -0.112 | | 0.079 | 0.156 | -0.122 | 0.066 | 0.065 | -0.073 | 0.060 | 0.223 | -0.088 | 0.070 | 0.209 |
| SMSCF T2 → MF T3 | -0.013 | | 0.089 | 0.881 | -0.041 | 0.117 | 0.726 | -0.073 | 0.060 | 0.223 | -0.088 | 0.070 | 0.209 |
| MF T1 → SMSCF T2 | -0.083 | | 0.218 | 0.703 | -0.180 | 0.194 | 0.353 | -0.216 | 0.169 | 0.203 | -0.270 | 0.194 | 0.165 |
| MF T2 → SMSCF T3 | -0.334 | | 0.189 | 0.077 | -0.383 | 0.229 | 0.094 | -0.216 | 0.169 | 0.203 | -0.270 | 0.194 | 0.165 |
| Covariance | | | | | | | | | | | | | |
| Between-person | -0.266 | | 0.042 | **0.000^***^** | -0.249 | 0.094 | **0.008^**^** | -0.263 | 0.073 | **0.000^***^** | -0.251 | 0.047 | **0.000^***^** |
| Intra-wave correlation T1 | -0.064 | | 0.038 | 0.096 | -0.082 | 0.090 | 0.363 | -0.064 | 0.070 | 0.360 | -0.080 | 0.036 | **0.028^*^** |
| Intra-wave correlation T2 | -0.068 | | 0.038 | 0.079 | -0.086 | 0.047 | 0.068 | -0.070 | 0.041 | 0.085 | -0.082 | 0.049 | 0.097 |
| Intra-wave correlation T3 | -0.045 | | 0.020 | **0.028^*^** | -0.045 | 0.022 | **0.044^*^** | -0.042 | 0.019 | **0.025^*^** | -0.040 | 0.020 | **0.042^*^** |
| Control variables |  | |  |  |  |  |  |  |  |  |  |  |  |
| SMSCF T1 → time | 0.201 | | 0.023 | **0.000^***^** | 0.199 | 0.023 | **0.000^***^** | 0.203 | 0.023 | **0.000^***^** | 0.200 | 0.023 | **0.000^***^** |
| SMSCF T2 → time | 0.201 | | 0.023 | **0.000^***^** | 0.199 | 0.023 | **0.000^***^** | 0.203 | 0.023 | **0.000^***^** | 0.200 | 0.023 | **0.000^***^** |
| SMSCF T3 → time | 0.201 | | 0.023 | **0.000^***^** | 0.199 | 0.023 | **0.000^***^** | 0.203 | 0.023 | **0.000^***^** | 0.200 | 0.023 | **0.000^***^** |
| MF T1 → time | -0.052 | | 0.022 | **0.020^*^** | -0.052 | 0.023 | **0.021^*^** | -0.053 | 0.022 | **0.019^*^** | -0.052 | 0.023 | **0.021^*^** |
| MF T2 → time | -0.052 | | 0.022 | **0.020^*^** | -0.052 | 0.023 | **0.021^*^** | -0.053 | 0.022 | **0.019^*^** | -0.052 | 0.023 | **0.021^*^** |
| MF T3 → time | -0.052 | | 0.022 | **0.020^*^** | -0.052 | 0.023 | **0.021^*^** | -0.053 | 0.022 | **0.019^*^** | -0.052 | 0.023 | **0.021^*^** |
| *N*_T1_ = 594, *N*_T2_ = 410, *N*_T3_ = 329, SMSCF = Social media self-control failure, MF = Mindfulness.  ^***^*p* < .001, ^**^*p* < .01, ^*^*p* < .05 | | | | | | | | | | | | | |

| **Table 5. Reciprocal relationships between social media self-control failure and subjective vitality adding age as a control variable** | | | | | | | | | | | | | |
| --- | --- | --- | --- | --- | --- | --- | --- | --- | --- | --- | --- | --- | --- |
|  | | Unconstrained model (*df = 9*) | | | Constrained autoregressive paths (*df = 7*) | | | Constrained cross-lagged paths (*df = 7*) | | | Constrained autoregressive and cross-lagged paths (*df = 9*) | | |
|  | | *b* | *SE* | *p* | *b* | *SE* | *p* | *b* | *SE* | *p* | *b* | *SE* | *p* |
| Autoregressive paths | | | | | | | | | | | | | |
| SMSCF T1 → SMSCF T2 | | -0.200 | 0.126 | 0.111 | -0.137 | 0.108 | 0.206 | -0.225 | 0.123 | 0.068 | -0.142 | 0.118 | 0.231 |
| SMSCF T2 → SMSCF T3 | | 0.028 | 0.131 | 0.830 | -0.137 | 0.108 | 0.206 | 0.025 | 0.143 | 0.859 | -0.142 | 0.118 | 0.231 |
| SVS T1 → SVS T2 | | 0.030 | 0.097 | 0.759 | -0.060 | 0.139 | 0.667 | -0.031 | 0.065 | 0.636 | -0.022 | 0.132 | 0.869 |
| SVS T2 → SVS T3 | | -0.516 | 0.326 | 0.113 | -0.060 | 0.139 | 0.667 | -0.376 | 0.419 | 0.369 | -0.022 | 0.132 | 0.869 |
| Cross-lagged paths | | | | | | | | | | | | | |
| SMSCF T1 → SVS T2 | 0.022 | | 0.069 | 0.744 | -0.040 | 0.094 | 0.671 | -0.031 | 0.065 | 0.636 | -0.059 | 0.064 | 0.353 |
| SMSCF T2 → SVS T3 | -0.234 | | 0.163 | 0.151 | -0.102 | 0.122 | 0.401 | -0.031 | 0.065 | 0.636 | -0.059 | 0.064 | 0.353 |
| SVS T1 → SMSCF T2 | -0.164 | | 0.145 | 0.258 | -0.144 | 0.159 | 0.366 | -0.106 | 0.147 | 0.468 | -0.218 | 0.149 | 0.144 |
| SVS T2 → SMSCF T3 | -0.173 | | 0.243 | 0.475 | -0.373 | 0.238 | 0.117 | -0.106 | 0.147 | 0.468 | -0.218 | 0.149 | 0.144 |
| Covariance | | | | | | | | | | | | | |
| Between-person | -0.158 | | 0.033 | **0.000^***^** | -0.150 | 0.036 | **0.000^***^** | -0.162 | 0.036 | **0.000^***^** | -0.146 | 0.036 | **0.000^***^** |
| Intra-wave correlation T1 | -0.015 | | 0.024 | 0.545 | -0.018 | 0.031 | 0.557 | -0.031 | 0.020 | 0.109 | -0.036 | 0.021 | 0.094 |
| Intra-wave correlation T2 | -0.059 | | 0.034 | 0.083 | -0.071 | 0.036 | 0.048 | -0.042 | 0.041 | 0.302 | -0.069 | 0.041 | 0.093 |
| Intra-wave correlation T3 | -0.041 | | 0.030 | 0.171 | -0.042 | 0.030 | 0.170 | -0.023 | 0.024 | 0.340 | -0.023 | 0.023 | 0.318 |
| Control variables |  | |  |  |  |  |  |  |  |  |  |  |  |
| SMSCF T1 → age | -0.026 | | 0.004 | **0.000^***^** | -0.025 | 0.004 | **0.000^***^** | -0.026 | 0.004 | **0.000^***^** | -0.025 | 0.004 | **0.000^***^** |
| SMSCF T2 → age | -0.026 | | 0.004 | **0.000^***^** | -0.025 | 0.004 | **0.000^***^** | -0.026 | 0.004 | **0.000^***^** | -0.025 | 0.004 | **0.000^***^** |
| SMSCF T3 → age | -0.026 | | 0.004 | **0.000^***^** | -0.025 | 0.004 | **0.000^***^** | -0.026 | 0.004 | **0.000^***^** | -0.025 | 0.004 | **0.000^***^** |
| SVS T1 → age | 0.000 | | 0.003 | 0.987 | -0.000 | 0.004 | 0.985 | -0.000 | 0.004 | 0.925 | -0.000 | 0.004 | 0.938 |
| SVS T2 → age | 0.000 | | 0.003 | 0.987 | -0.000 | 0.004 | 0.985 | -0.000 | 0.004 | 0.925 | -0.000 | 0.004 | 0.938 |
| SVS T3 → age | 0.000 | | 0.003 | 0.987 | -0.000 | 0.004 | 0.985 | -0.000 | 0.004 | 0.925 | -0.000 | 0.004 | 0.938 |
| *N*_T1_ = 594, *N*_T2_ = 410, *N*_T3_ = 329, SMSCF = Social media self-control failure, SVS = Subjective vitality scale.  ^***^*p* < .001, ^**^*p* < .01, ^*^*p* < .05 | | | | | | | | | | | | | |

| **Table 6. Reciprocal relationships between social media self-control failure and subjective vitality adding sex as a control variable** | | | | | | | | | | | | | |
| --- | --- | --- | --- | --- | --- | --- | --- | --- | --- | --- | --- | --- | --- |
|  | | Unconstrained model (*df = 9*) | | | Constrained autoregressive paths (*df = 7*) | | | Constrained cross-lagged paths (*df = 7*) | | | Constrained autoregressive and cross-lagged paths (*df = 9*) | | |
|  | | *b* | *SE* | *p* | *b* | *SE* | *p* | *b* | *SE* | *p* | *b* | *SE* | *p* |
| Autoregressive paths | | | | | | | | | | | | | |
| SMSCF T1 → SMSCF T2 | | -0.174 | 0.110 | 0.114 | -0.153 | 0.096 | 0.113 | -0.197 | 0.107 | 0.065 | -0.156 | 0.102 | 0.127 |
| SMSCF T2 → SMSCF T3 | | -0.065 | 0.174 | 0.709 | -0.153 | 0.096 | 0.113 | -0.058 | 0.158 | 0.713 | -0.156 | 0.102 | 0.127 |
| SVS T1 → SVS T2 | | 0.030 | 0.112 | 0.790 | -0.065 | 0.141 | 0.644 | 0.025 | 0.111 | 0.819 | -0.030 | 0.132 | 0.820 |
| SVS T2 → SVS T3 | | -0.494 | 0.424 | 0.243 | -0.065 | 0.141 | 0.644 | -0.349 | 0.394 | 0.375 | -0.030 | 0.132 | 0.820 |
| Cross-lagged paths | | | | | | | | | | | | | |
| SMSCF T1 → SVS T2 | 0.018 | | 0.072 | 0.797 | -0.024 | 0.092 | 0.796 | -0.031 | 0.062 | 0.619 | -0.050 | 0.059 | 0.395 |
| SMSCF T2 → SVS T3 | -0.260 | | 0.242 | 0.283 | -0.105 | 0.125 | 0.399 | -0.031 | 0.062 | 0.619 | -0.050 | 0.059 | 0.395 |
| SVS T1 → SMSCF T2 | -0.169 | | 0.149 | 0.256 | -0.130 | 0.165 | 0.429 | -0.118 | 0.142 | 0.405 | -0.199 | 0.132 | 0.130 |
| SVS T2 → SMSCF T3 | -0.218 | | 0.304 | 0.474 | -0.330 | 0.223 | 0.139 | -0.118 | 0.142 | 0.405 | -0.199 | 0.132 | 0.130 |
| Covariance | | | | | | | | | | | | | |
| Between-person | -0.150 | | 0.035 | **0.000^***^** | -0.147 | 0.036 | **0.000^***^** | -0.154 | 0.036 | **0.000^***^** | -0.142 | 0.035 | **0.000^***^** |
| Intra-wave correlation T1 | -0.014 | | 0.026 | 0.586 | -0.016 | 0.032 | 0.607 | -0.033 | 0.020 | 0.093 | -0.035 | 0.021 | 0.098 |
| Intra-wave correlation T2 | -0.061 | | 0.035 | 0.080 | -0.064 | 0.033 | **0.049** | -0.043 | 0.039 | 0.273 | -0.063 | 0.036 | 0.079 |
| Intra-wave correlation T3 | -0.043 | | 0.037 | 0.238 | -0.041 | 0.032 | 0.195 | -0.020 | 0.024 | 0.397 | -0.022 | 0.023 | 0.336 |
| Control variables |  | |  |  |  |  |  |  |  |  |  |  |  |
| SMSCF T1 → sex | 0.255 | | 0.086 | **0.003^**^** | 0.259 | 0.086 | **0.003^**^** | 0.253 | 0.086 | **0.003^**^** | 0.256 | 0.086 | **0.003^**^** |
| SMSCF T2 → sex | 0.255 | | 0.086 | **0.003^**^** | 0.259 | 0.086 | **0.003^**^** | 0.253 | 0.086 | **0.003^**^** | 0.256 | 0.086 | **0.003^**^** |
| SMSCF T3 → sex | 0.255 | | 0.086 | **0.003^**^** | 0.259 | 0.086 | **0.003^**^** | 0.253 | 0.086 | **0.003^**^** | 0.256 | 0.086 | **0.003^**^** |
| SVS T1 → sex | -0.143 | | 0.073 | **0.049^*^** | -0.157 | 0.072 | **0.029^*^** | -0.150 | 0.073 | **0.039^*^** | -0.157 | 0.072 | **0.029^*^** |
| SVS T2 → sex | -0.143 | | 0.073 | **0.049^*^** | -0.157 | 0.072 | **0.029^*^** | -0.150 | 0.073 | **0.039^*^** | -0.157 | 0.072 | **0.029^*^** |
| SVS T3 → sex | -0.143 | | 0.073 | **0.049^*^** | -0.157 | 0.072 | **0.029^*^** | -0.150 | 0.073 | **0.039^*^** | -0.157 | 0.072 | **0.029^*^** |
| *N*_T1_ = 594, *N*_T2_ = 410, *N*_T3_ = 329, SMSCF = Social media self-control failure, SVS = Subjective vitality scale.  ^***^*p* < .001, ^**^*p* < .01, ^*^*p* < .05 | | | | | | | | | | | | | |

| **Table 7. Reciprocal relationships between social media self-control failure and subjective vitality adding frequency of visit on social media as a control variable** | | | | | | | | | | | | | |
| --- | --- | --- | --- | --- | --- | --- | --- | --- | --- | --- | --- | --- | --- |
|  | | Unconstrained model (*df = 9*) | | | Constrained autoregressive paths (*df = 7*) | | | Constrained cross-lagged paths (*df = 7*) | | | Constrained autoregressive and cross-lagged paths (*df = 9*) | | |
|  | | *b* | *SE* | *p* | *b* | *SE* | *p* | *b* | *SE* | *p* | *b* | *SE* | *p* |
| Autoregressive paths | | | | | | | | | | | | | |
| SMSCF T1 → SMSCF T2 | | -0.153 | 0.113 | 0.176 | -0.114 | 0.105 | 0.277 | -0.174 | 0.112 | 0.122 | -0.112 | 0.113 | 0.320 |
| SMSCF T2 → SMSCF T3 | | -0.000 | 0.167 | 0.998 | -0.114 | 0.105 | 0.277 | 0.016 | 0.149 | 0.915 | -0.112 | 0.113 | 0.320 |
| SVS T1 → SVS T2 | | 0.041 | 0.111 | 0.710 | -0.056 | 0.139 | 0.689 | 0.039 | 0.111 | 0.724 | -0.016 | 0.132 | 0.902 |
| SVS T2 → SVS T3 | | -0.504 | 0.434 | 0.245 | -0.056 | 0.139 | 0.689 | -0.326 | 0.377 | 0.387 | -0.016 | 0.132 | 0.902 |
| Cross-lagged paths | | | | | | | | | | | | | |
| SMSCF T1 → SVS T2 | 0.000 | | 0.073 | 0.998 | -0.050 | 0.089 | 0.573 | -0.051 | 0.064 | 0.430 | -0.073 | 0.061 | 0.230 |
| SMSCF T2 → SVS T3 | -0.264 | | 0.234 | 0.260 | -0.109 | 0.112 | 0.330 | -0.051 | 0.064 | 0.430 | -0.073 | 0.061 | 0.230 |
| SVS T1 → SMSCF T2 | -0.196 | | 0.151 | 0.196 | -0.165 | 0.163 | 0.310 | -0.141 | 0.146 | 0.334 | -0.243 | 0.139 | 0.081 |
| SVS T2 → SMSCF T3 | -0.231 | | 0.288 | 0.422 | -0.373 | 0.212 | 0.079 | -0.141 | 0.146 | 0.334 | -0.243 | 0.139 | 0.081 |
| Covariance | | | | | | | | | | | | | |
| Between-person | -0.130 | | 0.035 | **0.000^***^** | -0.126 | 0.037 | **0.001^**^** | -0.134 | 0.037 | **0.000^***^** | -0.120 | 0.037 | **0.001^**^** |
| Intra-wave correlation T1 | -0.017 | | 0.026 | 0.512 | -0.020 | 0.031 | 0.516 | -0.034 | 0.021 | 0.097 | -0.037 | 0.022 | 0.091 |
| Intra-wave correlation T2 | -0.073 | | 0.035 | **0.040^*^** | -0.078 | 0.033 | **0.019^*^** | -0.055 | **0.039^*^** | 0.159 | -0.080 | 0.038 | **0.036^*^** |
| Intra-wave correlation T3 | -0.041 | | 0.034 | 0.223 | -0.039 | 0.028 | 0.165 | -0.020 | 0.023 | 0.370 | -0.023 | 0.022 | 0.311 |
| Control variables |  | |  |  |  |  |  |  |  |  |  |  |  |
| SMSCF T1 → frequency | 0.293 | | 0.032 | **0.000^***^** | 0.292 | 0.032 | **0.000^***^** | 0.293 | 0.032 | **0.000^***^** | 0.292 | 0.032 | **0.000^***^** |
| SMSCF T2 → frequency | 0.293 | | 0.032 | **0.000^***^** | 0.292 | 0.032 | **0.000^***^** | 0.293 | 0.032 | **0.000^***^** | 0.292 | 0.032 | **0.000^***^** |
| SMSCF T3 → frequency | 0.293 | | 0.032 | **0.000^***^** | 0.292 | 0.032 | **0.000^***^** | 0.293 | 0.032 | **0.000^***^** | 0.292 | 0.032 | **0.000^***^** |
| SVS T1 → frequency | -0.056 | | 0.031 | 0.068 | -0.054 | 0.031 | 0.076 | -0.054 | 0.031 | 0.075 | -0.054 | 0.030 | 0.077 |
| SVS T2 → frequency | -0.056 | | 0.031 | 0.068 | -0.054 | 0.031 | 0.076 | -0.054 | 0.031 | 0.075 | -0.054 | 0.030 | 0.077 |
| SVS T3 → frequency | -0.056 | | 0.031 | 0.068 | -0.054 | 0.031 | 0.076 | -0.054 | 0.031 | 0.075 | -0.054 | 0.030 | 0.077 |
| *N*_T1_ = 594, *N*_T2_ = 410, *N*_T3_ = 329, SMSCF = Social media self-control failure, SVS = Subjective vitality scale.  ^***^*p* < .001, ^**^*p* < .01, ^*^*p* < .05 | | | | | | | | | | | | | |

| **Table 8. Reciprocal relationships between social media self-control failure and subjective vitality adding time spent on social media as a control variable** | | | | | | | | | | | | | |
| --- | --- | --- | --- | --- | --- | --- | --- | --- | --- | --- | --- | --- | --- |
|  | | Unconstrained model (*df = 9*) | | | Constrained autoregressive paths (*df = 7*) | | | Constrained cross-lagged paths (*df = 7*) | | | Constrained autoregressive and cross-lagged paths (*df = 9*) | | |
|  | | *b* | *SE* | *p* | *b* | *SE* | *p* | *b* | *SE* | *p* | *b* | *SE* | *p* |
| Autoregressive paths | | | | | | | | | | | | | |
| SMSCF T1 → SMSCF T2 | | -0.210 | 0.115 | 0.068 | -0.158 | 0.101 | 0.116 | -0.223 | 0.112 | **0.047^*^** | -0.160 | 0.108 | 0.140 |
| SMSCF T2 → SMSCF T3 | | 0.018 | 0.157 | 0.910 | -0.158 | 0.101 | 0.116 | 0.008 | 0.148 | 0.959 | -0.160 | 0.108 | 0.140 |
| SVS T1 → SVS T2 | | 0.046 | 0.107 | 0.668 | -0.052 | 0.135 | 0.703 | 0.025 | 0.105 | 0.811 | -0.032 | 0.132 | 0.809 |
| SVS T2 → SVS T3 | | -0.510 | 0.407 | 0.210 | -0.052 | 0.135 | 0.703 | -0.392 | 0.374 | 0.294 | -0.032 | 0.132 | 0.809 |
| Cross-lagged paths | | | | | | | | | | | | | |
| SMSCF T1 → SVS T2 | 0.023 | | 0.069 | 0.736 | -0.027 | 0.088 | 0.763 | -0.033 | 0.057 | 0.560 | -0.051 | 0.059 | 0.391 |
| SMSCF T2 → SVS T3 | -0.251 | | 0.220 | 0.254 | -0.104 | 0.118 | 0.377 | -0.033 | 0.057 | 0.560 | -0.051 | 0.059 | 0.391 |
| SVS T1 → SMSCF T2 | -0.205 | | 0.141 | 0.148 | -0.168 | 0.151 | 0.263 | -0.118 | 0.128 | 0.358 | -0.200 | 0.137 | 0.143 |
| SVS T2 → SMSCF T3 | -0.117 | | 0.269 | 0.664 | -0.290 | 0.229 | 0.205 | -0.118 | 0.128 | 0.358 | -0.200 | 0.137 | 0.143 |
| Covariance | | | | | | | | | | | | | |
| Between-person | -0.135 | | 0.034 | **0.000^***^** | -0.132 | 0.035 | **0.000^***^** | -0.140 | 0.034 | **0.000^***^** | -0.129 | 0.035 | **0.000^***^** |
| Intra-wave correlation T1 | -0.021 | | 0.024 | 0.388 | -0.022 | 0.030 | 0.451 | -0.035 | 0.019 | 0.069 | -0.036 | 0.021 | 0.085 |
| Intra-wave correlation T2 | -0.060 | | 0.033 | 0.071 | -0.065 | 0.034 | 0.054 | -0.043 | 0.035 | 0.218 | -0.063 | 0.037 | 0.086 |
| Intra-wave correlation T3 | -0.031 | | 0.034 | 0.373 | -0.034 | 0.029 | 0.244 | -0.020 | 0.023 | 0.389 | -0.020 | 0.023 | 0.369 |
| Control variables |  | |  |  |  |  |  |  |  |  |  |  |  |
| SMSCF T1 → time | 0.207 | | 0.023 | **0.000^***^** | 0.204 | 0.023 | **0.000^***^** | 0.207 | 0.023 | **0.000^***^** | 0.205 | 0.023 | **0.000^***^** |
| SMSCF T2 → time | 0.207 | | 0.023 | **0.000^***^** | 0.204 | 0.023 | **0.000^***^** | 0.207 | 0.023 | **0.000^***^** | 0.205 | 0.023 | **0.000^***^** |
| SMSCF T3 → time | 0.207 | | 0.023 | **0.000^***^** | 0.204 | 0.023 | **0.000^***^** | 0.207 | 0.023 | **0.000^***^** | 0.205 | 0.023 | **0.000^***^** |
| SVS T1 → time | -0.045 | | 0.022 | **0.046^*^** | -0.041 | 0.022 | 0.065 | -0.043 | 0.022 | 0.054 | -0.040 | 0.022 | 0.070 |
| SVS T2→ time | -0.045 | | 0.022 | **0.046^*^** | -0.041 | 0.022 | 0.065 | -0.043 | 0.022 | 0.054 | -0.040 | 0.022 | 0.070 |
| SVS T3→ time | -0.045 | | 0.022 | **0.046^*^** | -0.041 | 0.022 | 0.065 | -0.043 | 0.022 | 0.054 | -0.040 | 0.022 | 0.070 |
| *N*_T1_ = 594, *N*_T2_ = 410, *N*_T3_ = 329, SMSCF = Social media self-control failure, SVS = Subjective vitality scale.  ^***^*p* < .001, ^**^*p* < .01, ^*^*p* < .05 | | | | | | | | | | | | | |

| **Table 9. Reciprocal relationships between social media self-control failure and satisfaction with life adding age as a control variable** | | | | | | | | | | | | | |
| --- | --- | --- | --- | --- | --- | --- | --- | --- | --- | --- | --- | --- | --- |
|  | | Unconstrained model (*df = 9*) | | | Constrained autoregressive paths (*df = 7*) | | | Constrained cross-lagged paths (*df = 7*) | | | Constrained autoregressive and cross-lagged paths (*df = 9*) | | |
|  | | *b* | *SE* | *p* | *b* | *SE* | *p* | *b* | *SE* | *p* | *b* | *SE* | *p* |
| Autoregressive paths | | | | | | | | | | | | | |
| SMSCF T1 → SMSCF T2 | | -0.148 | 0.114 | 0.195 | -0.104 | 0.097 | 0.286 | -0.147 | 0.111 | 0.185 | -0.093 | 0.100 | 0.351 |
| SMSCF T2 → SMSCF T3 | | -0.009 | 0.156 | 0.956 | -0.104 | 0.097 | 0.286 | 0.007 | 0.137 | 0.961 | -0.093 | 0.100 | 0.351 |
| SWLS T1 → SWLS T2 | | 0.208 | 0.176 | 0.237 | 0.178 | 0.147 | 0.229 | 0.222 | 0.166 | 0.182 | 0.214 | 0.127 | 0.091 |
| SWLS T2 → SWLS T3 | | 0.140 | 0.219 | 0.522 | 0.178 | 0.147 | 0.229 | 0.171 | 0.170 | 0.314 | 0.214 | 0.127 | 0.091 |
| Cross-lagged paths | | | | | | | | | | | | | |
| SMSCF T1 → SWLS T2 | -0.109 | | 0.116 | 0.345 | -0.131 | 0.113 | 0.244 | -0.127 | 0.081 | 0.120 | -0.144 | 0.082 | 0.080 |
| SMSCF T2 → SWLS T3 | -0.157 | | 0.202 | 0.438 | -0.146 | 0.164 | 0.371 | -0.127 | 0.081 | 0.120 | -0.144 | 0.082 | 0.080 |
| SWLS T1 → SMSCF T2 | -0.195 | | 0.125 | 0.121 | -0.203 | 0.116 | 0.081 | -0.206 | 0.096 | **0.032^*^** | -0.249 | 0.098 | **0.011^*^** |
| SWLS T2 → SMSCF T3 | -0.224 | | 0.152 | 0.140 | -0.298 | 0.133 | **0.025^*^** | -0.206 | 0.096 | **0.032^*^** | -0.249 | 0.098 | **0.011^*^** |
| Covariance | | | | | | | | | | | | | |
| Between-person | -0.122 | | 0.062 | **0.048^*^** | -0.116 | 0.062 | 0.059 | -0.119 | 0.061 | 0.051 | -0.109 | 0.060 | 0.070 |
| Intra-wave correlation T1 | 0.002 | | 0.047 | 0.963 | 0.003 | 0.048 | 0.951 | -0.006 | 0.035 | 0.857 | -0.010 | 0.034 | 0.774 |
| Intra-wave correlation T2 | -0.134 | | 0.049 | **0.007^**^** | -0.147 | 0.049 | **0.003^**^** | **-**0.135 | 0.049 | **0.006^**^** | -0.152 | 0.053 | **0.004^**^** |
| Intra-wave correlation T3 | -0.032 | | 0.036 | 0.374 | -0.033 | 0.034 | 0.331 | -0.027 | 0.031 | 0.390 | -0.023 | 0.030 | 0.449 |
| Control variables |  | |  |  |  |  |  |  |  |  |  |  |  |
| SMSCF T1 → age | -0.026 | | 0.004 | **0.000^***^** | -0.025 | 0.004 | **0.000^***^** | -0.026 | 0.004 | **0.000^***^** | -0.025 | 0.004 | **0.000^***^** |
| SMSCF T2 → age | -0.026 | | 0.004 | **0.000^***^** | -0.025 | 0.004 | **0.000^***^** | -0.026 | 0.004 | **0.000^***^** | -0.025 | 0.004 | **0.000^***^** |
| SMSCF T3 → age | -0.026 | | 0.004 | **0.000^***^** | -0.025 | 0.004 | **0.000^***^** | -0.026 | 0.004 | **0.000^***^** | -0.025 | 0.004 | **0.000^***^** |
| SWLS T1 → age | 0.001 | | 0.006 | 0.887 | 0.001 | 0.006 | 0.867 | 0.001 | 0.006 | 0.898 | 0.001 | 0.006 | 0.876 |
| SWLS T2 → age | 0.001 | | 0.006 | 0.887 | 0.001 | 0.006 | 0.867 | 0.001 | 0.006 | 0.898 | 0.001 | 0.006 | 0.876 |
| SWLS T3 → age | 0.001 | | 0.006 | 0.887 | 0.001 | 0.006 | 0.867 | 0.001 | 0.006 | 0.898 | 0.001 | 0.006 | 0.876 |
| *N*_T1_ = 594, *N*_T2_ = 410, *N*_T3_ = 329, SMSCF = Social media self-control failure, SWLS = Satisfaction with life scale.  ^***^*p* < .001, ^**^*p* < .01, ^*^*p* < .05 | | | | | | | | | | | | | |

| **Table 10. Reciprocal relationships between social media self-control failure and satisfaction with life adding sex as a control variable** | | | | | | | | | | | | | |
| --- | --- | --- | --- | --- | --- | --- | --- | --- | --- | --- | --- | --- | --- |
|  | | Unconstrained model (*df = 9*) | | | Constrained autoregressive paths (*df = 7*) | | | Constrained cross-lagged paths (*df = 7*) | | | Constrained autoregressive and cross-lagged paths (*df = 9*) | | |
|  | | *b* | *SE* | *p* | *b* | *SE* | *p* | *b* | *SE* | *p* | *b* | *SE* | *p* |
| Autoregressive paths | | | | | | | | | | | | | |
| SMSCF T1 → SMSCF T2 | | 0.136 | 0.102 | 0.182 | -0.124 | 0.091 | 0.174 | -0.138 | 0.100 | 0.167 | -0.121 | 0.092 | 0.190 |
| SMSCF T2 → SMSCF T3 | | -0.091 | 0.172 | 0.598 | -0.124 | 0.091 | 0.174 | -0.073 | 0.147 | 0.617 | -0.121 | 0.092 | 0.190 |
| SWLST1 → SWLS T2 | | 0.212 | 0.174 | 0.222 | 0.184 | 0.149 | 0.215 | 0.222 | 0.168 | 0.187 | 0.202 | 0.134 | 0.132 |
| SWLS T2 → SWLS T3 | | 0.145 | 0.216 | 0.501 | 0.184 | 0.149 | 0.215 | 0.171 | 0.176 | 0.330 | 0.202 | 0.134 | 0.132 |
| Cross-lagged paths | | | | | | | | | | | | | |
| SMSCF T1 → SWLS T2 | -0.110 | | 0.110 | 0.315 | -0.118 | 0.111 | 0.288 | -0.121 | 0.081 | 0.137 | -0.120 | 0.081 | 0.139 |
| SMSCF T2 → SWLS T3 | -0.149 | | 0.214 | 0.487 | -0.117 | 0.170 | 0.494 | -0.121 | 0.081 | 0.137 | -0.120 | 0.081 | 0.139 |
| SWLS T1 → SMSCF T2 | -0.206 | | 0.118 | 0.081 | -0.203 | 0.116 | 0.081 | -0.216 | 0.094 | **0.022^*^** | -0.228 | 0.092 | **0.014^*^** |
| SWLS T2 → SMSCF T3 | -0.236 | | 0.163 | 0.148 | -0.258 | 0.135 | 0.056 | -0.216 | 0.094 | **0.022^*^** | -0.228 | 0.092 | **0.014^*^** |
| Covariance | | | | | | | | | | | | | |
| Between-person | -0.117 | | 0.063 | 0.062 | -0.117 | 0.063 | 0.062 | -0.115 | 0.062 | 0.065 | -0.114 | 0.061 | 0.061 |
| Intra-wave correlation T1 | -0.008 | | 0.047 | 0.864 | -0.008 | 0.049 | 0.872 | -0.015 | 0.036 | 0.676 | -0.014 | 0.034 | 0.685 |
| Intra-wave correlation T2 | -0.133 | | 0.049 | **0.006^**^** | -0.135 | 0.048 | **0.005^**^** | **-**0.133 | 0.049 | **0.007^**^** | -0.136 | 0.050 | **0.007^**^** |
| Intra-wave correlation T3 | -0.024 | | 0.037 | 0.524 | -0.022 | 0.035 | 0.531 | -0.019 | 0.031 | 0.552 | -0.016 | 0.030 | 0.599 |
| Control variables |  | |  |  |  |  |  |  |  |  |  |  |  |
| SMSCF T1 → sex | 0.241 | | 0.087 | **0.005^**^** | 0.242 | 0.087 | **0.005^**^** | 0.241 | 0.086 | **0.005^**^** | 0.242 | 0.086 | **0.005^**^** |
| SMSCF T2 → sex | 0.241 | | 0.087 | **0.005^**^** | 0.242 | 0.087 | **0.005^**^** | 0.241 | 0.086 | **0.005^**^** | 0.242 | 0.086 | **0.005^**^** |
| SMSCF T3 → sex | 0.241 | | 0.087 | **0.005^**^** | 0.242 | 0.087 | **0.005^**^** | 0.241 | 0.086 | **0.005^**^** | 0.242 | 0.086 | **0.005^**^** |
| SVS T1 → sex | -0.022 | | 0.127 | 0.863 | -0.023 | 0.126 | 0.855 | -0.021 | 0.126 | 0.867 | -0.021 | 0.126 | 0.868 |
| SWLS T2 → sex | -0.022 | | 0.127 | 0.863 | -0.023 | 0.126 | 0.855 | -0.021 | 0.126 | 0.867 | -0.021 | 0.126 | 0.868 |
| SWLS T3 → sex | -0.022 | | 0.127 | 0.863 | -0.023 | 0.126 | 0.855 | -0.021 | 0.126 | 0.867 | -0.021 | 0.126 | 0.868 |
| *N*_T1_ = 594, *N*_T2_ = 410, *N*_T3_ = 329, SMSCF = Social media self-control failure, SWLS = Satisfaction with life scale.  ^***^*p* < .001, ^**^*p* < .01, ^*^*p* < .05 | | | | | | | | | | | | | |

| **Table 11. Reciprocal relationships between social media self-control failure and satisfaction with life adding frequency of visit on social media as a control variable** | | | | | | | | | | | | | |
| --- | --- | --- | --- | --- | --- | --- | --- | --- | --- | --- | --- | --- | --- |
|  | | Unconstrained model (*df = 9*) | | | Constrained autoregressive paths (*df = 7*) | | | Constrained cross-lagged paths (*df = 7*) | | | Constrained autoregressive and cross-lagged paths (*df = 9*) | | |
|  | | *b* | *SE* | *p* | *b* | *SE* | *p* | *b* | *SE* | *p* | *b* | *SE* | *p* |
| Autoregressive paths | | | | | | | | | | | | | |
| SMSCF T1 → SMSCF T2 | | -0.104 | 0.104 | 0.317 | -0.080 | 0.095 | 0.401 | -0.105 | 0.102 | 0.300 | -0.072 | 0.096 | 0.453 |
| SMSCF T2 → SMSCF T3 | | -0.027 | 0.163 | 0.870 | -0.080 | 0.095 | 0.401 | -0.006 | 0.142 | 0.965 | -0.072 | 0.096 | 0.453 |
| SWLS T1 → SWLS T2 | | 0.205 | 0.176 | 0.244 | 0.177 | 0.149 | 0.236 | 0.225 | 0.166 | 0.176 | 0.207 | 0.127 | 0.102 |
| SWLS T2 → SWLS T3 | | 0.139 | 0.220 | 0.526 | 0.177 | 0.149 | 0.236 | 0.171 | 0.169 | 0.311 | 0.207 | 0.127 | 0.102 |
| Cross-lagged paths | | | | | | | | | | | | | |
| SMSCF T1 → SWLS T2 | -0.130 | | 0.106 | 0.221 | -0.143 | 0.106 | 0.178 | -0.140 | 0.077 | 0.069 | -0.148 | 0.077 | 0.053 |
| SMSCF T2 → SWLS T3 | -0.156 | | 0.205 | 0.448 | -0.136 | 0.161 | 0.396 | -0.140 | 0.077 | 0.069 | -0.148 | 0.077 | 0.053 |
| SWLS T1 → SMSCF T2 | -0.198 | | 0.125 | 0.112 | -0.202 | 0.118 | 0.088 | -0.218 | 0.092 | **0.018^*^** | -0.248 | 0.091 | **0.006^**^** |
| SWLS T2 → SMSCF T3 | -0.251 | | 0.149 | 0.092 | -0.294 | 0.126 | **0.020^*^** | -0.218 | 0.092 | **0.018^*^** | -0.248 | 0.091 | **0.006^**^** |
| Covariance | | | | | | | | | | | | | |
| Between-person | -0.108 | | 0.061 | 0.075 | -0.105 | 0.060 | 0.082 | -0.104 | 0.059 | 0.078 | -0.099 | 0.058 | 0.089 |
| Intra-wave correlation T1 | -0.002 | | 0.047 | 0.964 | -0.002 | 0.049 | 0.969 | -0.011 | 0.036 | 0.769 | -0.012 | 0.034 | 0.734 |
| Intra-wave correlation T2 | -0.142 | | 0.048 | **0.003^**^** | -0.148 | 0.047 | **0.002^**^** | -0.143 | 0.046 | **0.002^**^** | -0.154 | 0.048 | **0.002^**^** |
| Intra-wave correlation T3 | -0.033 | | 0.036 | 0.361 | -0.032 | 0.034 | 0.344 | -0.026 | 0.031 | 0.396 | -0.024 | 0.030 | 0.431 |
| Control variables |  | |  |  |  |  |  |  |  |  |  |  |  |
| SMSCF T1 → frequency | 0.293 | | 0.032 | **0.000^***^** | 0.292 | 0.032 | **0.000^***^** | 0.293 | 0.032 | **0.000^***^** | 0.292 | 0.032 | **0.000^***^** |
| SMSCF T2 → frequency | 0.293 | | 0.032 | **0.000^***^** | 0.292 | 0.032 | **0.000^***^** | 0.293 | 0.032 | **0.000^***^** | 0.292 | 0.032 | **0.000^***^** |
| SMSCF T3 → frequency | 0.293 | | 0.032 | **0.000^***^** | 0.292 | 0.032 | **0.000^***^** | 0.293 | 0.032 | **0.000^***^** | 0.292 | 0.032 | **0.000^***^** |
| SWLS T 1 → frequency | -0.020 | | 0.051 | 0.692 | -0.021 | 0.050 | 0.684 | -0.020 | 0.050 | 0.691 | -0.021 | 0.050 | 0.675 |
| SWLS T2 → frequency | -0.020 | | 0.051 | 0.692 | -0.021 | 0.050 | 0.684 | -0.020 | 0.050 | 0.691 | -0.021 | 0.050 | 0.675 |
| SWLS T3 → frequency | -0.020 | | 0.051 | 0.692 | -0.021 | 0.050 | 0.684 | -0.020 | 0.050 | 0.691 | -0.021 | 0.050 | 0.675 |
| *N*_T1_ = 594, *N*_T2_ = 410, *N*_T3_ = 329, SMSCF = Social media self-control failure, SWLS = Satisfaction with life scale.  ^***^*p* < .001, ^**^*p* < .01, ^*^*p* < .05 | | | | | | | | | | | | | |

| **Table 12. Reciprocal relationships between social media self-control failure and satisfaction with life scale adding time spent on social media as a control variable** | | | | | | | | | | | | | |
| --- | --- | --- | --- | --- | --- | --- | --- | --- | --- | --- | --- | --- | --- |
|  | | Unconstrained model (*df = 9*) | | | Constrained autoregressive paths (*df = 7*) | | | Constrained cross-lagged paths (*df = 7*) | | | Constrained autoregressive and cross-lagged paths (*df = 9*) | | |
|  | | *b* | *SE* | *p* | *b* | *SE* | *p* | *b* | *SE* | *p* | *b* | *SE* | *p* |
| Autoregressive paths | | | | | | | | | | | | | |
| SMSCF T1 → SMSCF T2 | | -0.164 | 0.112 | 0.143 | -0.122 | 0.096 | 0.202 | -0.160 | 0.109 | 0.139 | -0.107 | 0.099 | 0.278 |
| SMSCF T2 → SMSCF T3 | | -0.014 | 0.160 | 0.932 | -0.122 | 0.096 | 0.202 | 0.001 | 0.144 | 0.994 | -0.107 | 0.099 | 0.278 |
| SWLS T1 → SWLS T2 | | 0.189 | 0.179 | 0.293 | 0.159 | 0.149 | 0.285 | 0.201 | 0.175 | 0.249 | 0.212 | 0.138 | 0.125 |
| SWLS T2 → SWLS T3 | | 0.127 | 0.222 | 0.566 | 0.159 | 0.149 | 0.285 | 0.178 | 0.180 | 0.323 | 0.212 | 0.138 | 0.125 |
| Cross-lagged paths | | | | | | | | | | | | | |
| SMSCF T1 → SWLS T2 | -0.061 | | 0.118 | 0.602 | -0.082 | 0.115 | 0.478 | -0.103 | 0.083 | 0.216 | -0.125 | 0.086 | 0.144 |
| SMSCF T2 → SWLS T3 | -0.179 | | 0.204 | 0.381 | -0.180 | 0.171 | 0.293 | -0.103 | 0.083 | 0.216 | -0.125 | 0.086 | 0.144 |
| SWLS T1 → SMSCF T2 | -0.181 | | 0.125 | 0.149 | -0.190 | 0.116 | 0.100 | -0.181 | 0.097 | 0.063 | -0.225 | 0.102 | **0.027^*^** |
| SWLS T2 → SMSCF T3 | -0.185 | | 0.153 | 0.225 | -0.261 | 0.140 | 0.063 | -0.181 | 0.097 | 0.063 | -0.225 | 0.102 | **0.027^*^** |
| Covariance | | | | | | | | | | | | | |
| Between-person | -0.100 | | 0.060 | 0.093 | -0.094 | 0.059 | 0.112 | -0.096 | 0.059 | 0.103 | -0.085 | 0.059 | 0.152 |
| Intra-wave correlation T1 | 0.010 | | 0.045 | 0.833 | 0.012 | 0.046 | 0.800 | -0.004 | 0.034 | 0.912 | -0.009 | 0.034 | 0.798 |
| Intra-wave correlation T2 | -0.119 | | 0.050 | **0.017^*^** | -0.132 | 0.050 | **0.009^**^** | -0.119 | 0.050 | **0.018^*^** | -0.137 | 0.055 | **0.013^*^** |
| Intra-wave correlation T3 | -0.033 | | 0.037 | 0.370 | -0.035 | 0.035 | 0.315 | -0.026 | 0.031 | 0.398 | -0.023 | 0.031 | 0.450 |
| Control variables |  | |  |  |  |  |  |  |  |  |  |  |  |
| SMSCF T1 → time | 0.205 | | 0.023 | **0.000^***^** | 0.203 | 0.023 | **0.000^***^** | 0.204 | 0.023 | **0.000^***^** | 0.202 | 0.022 | **0.000^***^** |
| SMSCF T2 → time | 0.205 | | 0.023 | **0.000^***^** | 0.203 | 0.023 | **0.000^***^** | 0.204 | 0.023 | **0.000^***^** | 0.202 | 0.022 | **0.000^***^** |
| SMSCF T3 → time | 0.205 | | 0.023 | **0.000^***^** | 0.203 | 0.023 | **0.000^***^** | 0.204 | 0.023 | **0.000^***^** | 0.202 | 0.022 | **0.000^***^** |
| SWLS T1 → time | -0.062 | | 0.037 | 0.098 | -0.063 | 0.037 | 0.094 | -0.061 | 0.037 | 0.102 | -0.061 | 0.037 | 0.102 |
| SWLS T2 → time | -0.062 | | 0.037 | 0.098 | -0.063 | 0.037 | 0.094 | -0.061 | 0.037 | 0.102 | -0.061 | 0.037 | 0.102 |
| SWLS T3 → time | -0.062 | | 0.037 | 0.098 | -0.063 | 0.037 | 0.094 | -0.061 | 0.037 | 0.102 | -0.061 | 0.037 | 0.102 |
| *N*_T1_ = 594, *N*_T2_ = 410, *N*_T3_ = 329, SMSCF = Social media self-control failure, SWLS = Satisfaction with life scale.  ^***^*p* < .001, ^**^*p* < .01, ^*^*p* < .05 | | | | | | | | | | | | | |
