# Supplementary material for: The reciprocal relationships between social media self-control failure, mindfulness and wellbeing: A longitudinal study
Source: PLoS One. 2021 Aug 4;16(8):e0255648. doi: 10.1371/journal.pone.0255648 (PMC8336798; doi:10.1371/journal.pone.0255648)
Supplement: Attachment — Submitted filename: Response letter.docx [file pone.0255648.s004.docx]

**Reviewer #1: I already liked the first version of the manuscript and I think you did an outstanding job on revising the manuscript, carrying out additional helpful analyses, and crafting the response letter in a generally convincing and well-organized way.**

Response to reviewer 1:

We would like to thank reviewer 1 for the positive feedback on our manuscript. We are grateful for the feedback because we feel that this improved the quality of the manuscript.

**I was convinced by most of your answers. Nevertheless, some minor clarifications would help further improve the paper.
1. Regarding my previous Point 2, thanks for uploading the codebook, the data, and the R code. I ran the code and it generally worked well (but see 1a and 1b for necessary modifications).
a) To foster reproducibility, it would help to include some instructions or lines of code how to load the data before running the code, for instance, in a readme file or, even better, directly within the code file.**

Response to reviewer 1 comment 1-a:

Following the suggestion of reviewer 1, we have added a line regarding how to locate the original data:

“#Read data, between the brackets should be the local location of the raw data on your device

merge <- read.csv("~/Desktop/PlosOne/Revision/7 Data analysis (study 2)/20190712 Data analysis/20190712/merge.csv")”

**b) Please insert that the tidyverse (or similar) package needs to be loaded. Otherwise, the %>% cannot be interpreted properly.**

Response to reviewer 1 comment 1-b:

Thank you for pointing this out. We have now added the dplyr package in the R script: “library(dplyr)”

**c) The sessionInfo() makes only sense if its output is included in a static file (e.g., a html output of an RMarkdown document). Only then, it will include the necessary info to reproduce the specific environment of your analyses. Otherwise, it will just produce the info of the session the reproducer ran.**

Response to reviewer 1 comment 1-c:

To solve this problem, we now directly copied the output information of the sessioninfo() at the beginning of the R script. We also added the sessioninfo output in the Rmarkdown html output.

**d) To view your analyses and results, a static output file (e.g., a html output of an RMarkdown document) may be of great benefit to the readers as well, because they don’t have to carry out the cumbersome bootstrapping procedures for all the models but can just look up what you’ve done and what the output looks like.**

Response to reviewer 1 comment 1-d:

Thank you for this helpful advice. We have now created an RMarkdown document in html version to store all scripts and results. The file can be found at the Open Science Framework <https://osf.io/hzy8r/> We refer to this file on p. 11 in the revised version of our manuscript.

**2. Ad 2b), it’s great that you’ve conducted additional analyses including control variables and integrated the new findings. Here, three minor issues arise:
a) You should cite your own findings as a reason why you’ve included exactly these control variables (Du et al., 2019).**

Response to reviewer 1 comment 2-a:

Following the suggestion of the reviewer, we now refer to two previous studies that showed that age, sex and social media use were related to social media self-control failure as a reason to include these as control variables. The revised paragraph now reads as follows (pp. 20):

“In order to check for the robustness of the results, in an exploratory analysis we added age, gender, and social media use (both the frequency of visit on social media and time spent on social media) as control variables to the model. The control variables were selected based on the findings in other studies, which showed that age was positively related to social media self-control failure, social media use was negatively related to social media self-control failure, and female participants tend to report higher levels of social media self-control failure than male participants [2][7].”

**b) If I’m not mistaken, Mulder and Hamaker (2020) is not yet listed in the references of your paper.**

Response to reviewer 1 comment 2-b:

Thank you for pointing this out; we now added Mulder and Hamaker (2020) to the references.

**c) Compared to the paper’s tables, the S2 Appendix is a bit awkward to read. I recommend a more straightforward depiction of the relevant findings to make it easier for the readers to compare the paths across models.**

Response to reviewer 1 comment 2-c:

We agree with reviewer 1 that we could improve the readability of the results in S2. Thus, to compare the paths across different models with each of the original model, we described the relevant findings in paragraphs in the manuscript, and also mentioned S2 file as a reference. We now also presented the results in tables to make it easier for the readers (see S2 file for more information). The paragraphs are listed below:

SMSCF-Mindfulness (pp. 21):

“Regarding the associations of the trait-like individual differences between the two variables, the results remained unchanged after controlling for age, gender and time spent on social media. Yet, adding frequency of visiting social media to the model did affect the results which in one the four models (the models which constrained the cross-lagged and autoregressive paths) were no longer significant.

Regarding the within-person change of the two variables, adding age resulted in a full reciprocal effect between social media self-control failure and mindfulness in the model which constrained both autoregressive and cross-lagged paths to be equal. Social media self-control failure predicted lower mindfulness and vice versa. Yet, after controlling for gender or frequency of visit, the prediction from social media self-control failure T1 to mindfulness T2 were no longer significant in the unconstraint model. After controlling for time spent on social media, the cross-lagged paths were no longer significant.”

SMSCF-Subjective Vitality (pp. 23):

“Controlling for adding age, gender, frequency of visit on social media and time spent on social media as control variables did not affect any of the results.”

SMSCF-Life satisfaction (p. 25):

“Controlling for age, gender, frequency of visit on social media, and time spent on social media did not affect the results regarding the associations between the trait-like individual differences.

The results regarding within-person change remained the same after controlling for age and frequency of visit on social media. However, after controlling for gender, in the model which constrained the autoregressive paths to be equal, there was no longer a significant path from life satisfaction T2 to social media self-control failure T3. Also, controlling for time spent on social media rendered the paths from life satisfaction T1 to social media self-control failure T2, and from T2 to T3 were no longer significant in the model that constrained the cross-lagged paths.”

**3. The only point where I disagree with you concerns 2a and Reviewer 2’s fifth point.
a) I don’t think that referring to “common practice” is a sound argument for not moving into a latent variables framework. On the same basis, you could have just carried out a traditional cross-lagged model. You would have found even more studies doing so but that not implies that this is the best or appropriate way to do it.**

Response to reviewer 1 comment 3-a:

Referring to common practice may indeed not be the best argument. However, the fact that using latent variables has only recently been introduced in RI-CLPM makes for a situation where solutions for problems you may run into have not yet been provided. We have tried a model with latent variables, following the 4-step process suggested by Mulder and Hamaker (2020). However, the results showed estimation problems during each step. For instance, regarding the model of social media self-control failure and mindfulness, the configural model showed that the model converged, but not all elements of the gradient are zero; The weak factorial invariance model showed that the covariance matrix of latent variables is not positive definite; The strong factorial invariance model and the strong factorial invariance model with factor loadings equal to the within-person factor loadings showed that the variance-covariance matrix of the estimated parameters was not positive definite. Due to this issue, some regression paths were not successfully estimated in each of the steps.

Considering the high number of items (15 items in total) in the Mindfulness Attention Awareness scale, we assessed whether the estimation problems were due to problems in the scale by testing a model using only the first 4 items to estimate the model. This model was successfully estimated, indicating that part of the problems were indeed due to the MAAS. This made us consider item parceling as a solution, however in the RI-CLPM this creates issues in the part of the model where we specify the random intercepts for each indicator separately (and in other parts as well).

In the other models we also run into estimation issues which we don’t know how to properly solve. Therefore, we feel that using latent variables brings us into uncharted territory. Solutions may exist, but given how recently latent variable RI-CLPM has been introduced, we cannot rely on the robustness and correctness of such solutions. Therefore, we prefer to hold on to using mean scores as indicators in our models.

To discuss the issue raised by Reviewer 1, we have added the following text to the discussion (pp. 39):

“Besides, the current research is limited by using mean score of each variable to fit the RI-CLPM, based on the rather strong assumption of no measurement error. However, this could bias lagged-parameter estimates, which weakens the explanatory power of the findings [55]. We also tried the latent variable model in analyzing the data. Following Hamaker & Mulder (2020), estimating the latent model is a 4-step process which helps build measurement invariance. However, results showed estimation problems during each step, which made us decide to hold on to using mean scores as indicators in our models. Future research could use multiple indicators of each variable to fit the RI-CLPM, or use alternative reciprocal models that account for measurement error, such as the stable trait autoregressive trait and state model (see Usami et al. (2019) for a review of different types of reciprocal models) [64].”

**b) I also don’t buy that there’s little practice in moving to a latent framework. You’ve already drawn on Mulder and Hamaker (2020) to include the control variables. They also wrote a section on the multiple-indicator RI-CLPM. Ironically, the “common” RI-CLPM is a special case of the more general multiple-indicator RI-CPLM and both are closely connected to latent growth curves with structured residuals (Thomas et al., 2021; Usami et al., 2019; Zyphur, Allison, et al., 2019; Zyphur, Voelkle, et al., 2019).**

Response to reviewer 1 comment 3-b:

Please see our response to 3-a.

**c) Similar to Reviewer 2, please don’t get me wrong: I don’t urge you to move to a latent variables framework if you have good reasons to not do so. From my perspective, however, the reasons you’ve mentioned thus far are not enough to convince me. Moreover, the CFAs reported in S3 rather obscure than illuminate the factorial validity of the constructs. For instance, for three-item-single-factor models you have to impose constraints to make them identifiable. I recommend running multi-construct latent state–trait models (Eid et al., 1994; Geiser, 2020; Steyer et al., 2015) or, at least, CFAs including all latent constructs of interest at the same measurement occasion to examine factorial structure and potential cross-loadings.**
Response to reviewer 1 comment 3-c:

Regarding the discussion about latent RI-CLPM, please see our response to 3-a. Regarding the CFA results, following the suggestion of Reviewer 1, we have now included all latent constructs of interest at each time in the same measurement occasion to examine the factorial structure. The results are reported in the S3 file.

**Reviewer #2: Dear authors,**

**Thank you for the opportunity to read this manuscript again. I liked the original admission and am impressed by the work you invested into the revision. The paper has become even more coherent and you addressed all concerns that I had (and, from my reading, the other reviewer had). My compliments. I think your work will make a valuable contribution to the literature and I look forward to seeing it in print.**

**1 Below just a couple of random thoughts that I DON’T want you to address in a second round, but wanted to share anyway:
• “****Empirical evidence confirmed” ◊ I’d prefer “support”
• One more sentence on the possible mechanism self-control failure ◊ lower mindfulness would’ve been helpful. Something like: “Repeatedly failing to respond to affective cues, thereby experiencing self-control failure, might fur[=ther decrease people’s sensitivity to those cues over time. In other words, self-control failure ◊ lower mindfulness”. Just a suggestion for future papers, no need to implement here. Actually, you have a similar sentence in the discussion.
• Really strong justification for distinction of trait vs. state.
• Exemplary transparency on reporting the first study based on these data.
• I’m not convinced bootstrapping solves the family-wise error rate. But I’m not a statistician, so I need to read up on this.
• Again: Exemplary treatment of the falsification criteria.
• I’m personally skeptical of the mindfulness interventions (see** [**https://doi.org/10.1177/1745691617709589**](https://doi.org/10.1177/1745691617709589)**), but not sure that criticism affects your conclusions about mindfulness treatments in the discussion.**

Response to reviewer 2 comment 1:

We thank reviewer 2 for the positive feedback and the suggestions. Following the suggestion of reviewer 2, we have changed the word “Empirical evidence confirmed” into “Empirical evidence supported”, and added one sentence to further explain the relationship between social media self-control failure and mindfulness. We thank the reviewer for sharing his thoughts regarding the other issues and we will keep these in mind for future papers.

**2. A couple of thoughts (independent of the content of the paper) on reproducibility, which can be addressed within half an hour:
1)• I tried reproducing your analysis: There’s no “DurationT1” variable in the data set.
2)• You use dplyr pipes, but don’t load any tidyverse packages.
3)• It would be ideal if you tried running the entire script from beginning to end on a new machine to check whether results can be reproduced.
4)• The output of sessionInfo() as a text file should ideally be added to the project for reproducibility.
5)• I had to request access to the OSF project – can you remove me again as a collaborator?
One more time: My compliments and congratulations on your work!
Niklas Johannes**

Response to reviewer 2 comment 2-1:

Regarding the variable “DurationT1”, this variable name could be generated after changing the name of the raw data (the original variable name in the raw data is “Duration_in_seconds”). The corresponding R code is: colnames(merge)[colnames(merge)=="Duration__in_seconds"] <- "DurationT1".

Response to reviewer 2 comment 2-2:

We have also added a line to load dplyr package. Please also refer to reviewer 1 response 1-b, 1-c and 1-d.

Response to reviewer 2 comment 2-3 and 2-4:

We have re-run all scripts, and also created a Rmarkdown html output file, the script, as well as all results are now stored in this output file, including the sessioninfo.

Response to reviewer 2 comment 2-5:

We have now removed you from the collaborator list.
